# Affinity Membranes and Monoliths for Protein Purification

**DOI:** 10.3390/membranes10010001

**Published:** 2019-12-24

**Authors:** Eleonora Lalli, Jouciane S. Silva, Cristiana Boi, Giulio C. Sarti

**Affiliations:** Dipartimento di Ingegneria Civile, Chimica, Ambientale e dei Materiali, DICAM, Alma Mater Studiorum Università di Bologna, via Terracini 28, 40131 Bologna, Italy; eleonora.lalli2@unibo.it (E.L.); jouciane@gmail.com (J.S.S.); giulio.sarti@unibo.it (G.C.S.)

**Keywords:** affinity chromatography, beads, membranes, monoliths, surface modification, proteins

## Abstract

Affinity capture represents an important step in downstream processing of proteins and it is conventionally performed through a chromatographic process. The performance of this step highly depends on the type of matrix employed. In particular, resin beads and convective materials, such as membranes and monoliths, are the commonly available supports. The present work deals with non-competitive binding of bovine serum albumin (BSA) on different chromatographic media functionalized with Cibacron Blue F3GA (CB). The aim is to set up the development of the purification process starting from the lab-scale characterization of a commercially available CB resin, regenerated cellulose membranes and polymeric monoliths, functionalized with CB to identify the best option. The performance of the three different chromatographic media is evaluated in terms of BSA binding capacity and productivity. The experimental investigation shows promising results for regenerated cellulose membranes and monoliths, whose performance are comparable with those of the packed column tested. It was demonstrated that the capacity of convective stationary phases does not depend on flow rate, in the range investigated, and that the productivity that can be achieved with membranes is 10 to 20 times higher depending on the initial BSA concentration value, and with monoliths it is approximately twice that of beads, at the same superficial velocity.

## 1. Introduction

Selectivity, versatility and efficiency make chromatography a widely used technique for protein purification and separation, due to the great number of possible chemical interactions that intervene between the target biomolecule and the stationary phase and to the availability of many adsorbent materials. Among the different kind of chromatographic separations usually needed for recovery and purification of a particular molecule, the main step in downstream processing is represented by the affinity capture. It allows the selective and efficient recovery of the target molecule thanks to a specific and reversible interaction between a ligand, immobilized on the support material, and the product itself.

Affinity chromatography is the most largely used separation technique for the purification and recovery of highly valuable biomolecules, such as enzymes, hormones, vaccines, DNA and RNA fragments and monoclonal antibodies [1,2]. Columns packed with functionalized resins or polymeric matrices, such as agarose, in the shape of spherical beads, represent the equipment traditionally used. Affinity particles are typically 50 to 100 μm in diameter, to minimize pressure drops [3] and the main mass transport mechanism toward the binding sites is diffusion [4]. Columns packed with chromatographic resins exhibit a good performance in terms of binding capacity, thanks to their high surface area, but the functionalized materials are usually very expensive and several limitations, such as high pressure drops, compaction of the packed bed, and slow intraparticle diffusion, lead to low productivity, poor ligand usage and long process time [5,6,7]. Consequently, the volume of elution buffers increases and biomolecules might be degraded because of long exposure to aggressive conditions. On the other hand, the use of smaller particles, with greater superficial area and shorter diffusive distance, has the drawback of drastically increasing the pressure drops and thus the operative costs to an extent that makes the separation process unprofitable [8]. Finally, the complex description of the transport phenomena in packed columns translates into a complicated scale-up process [9].

The use of porous chromatographic materials where the main mass transport mechanism toward the binding sites is convection can overcome the limitations that affect packed columns [10], especially reducing process time [11,12] and avoiding biomolecule degradation [13,14]. Convective chromatographic materials are represented by microporous membranes and monoliths.

Commonly employed materials for chromatographic membrane production are cellulose, regenerated cellulose, nylon, polyethylene and polyamide [15]. The use of membranes eliminates packing requirements and allows the registration of lower pressure drops, making the process less costly; it is thus possible to work at higher flow rates reducing process time. Membrane chromatography is particularly indicated for the capture of large biomolecules, like antibodies, viruses, DNA fragments, endotoxins and host cell proteins [16,17,18]. Different studies have been carried out to demonstrate the superior performance of affinity membranes when compared to chromatographic beads [19,20,21] in the field of protein purification. However, membranes are usually characterized by a lower specific area that translates into lower capacity [22] than that of porous beads, and an increase of that value may reduce pore sizes, increase pressure drop and lead to a nonuniform pore size distribution.

Monolithic supports and membranes have similar pore morphology, but they have different manufacturing technology [23,24]. A commonly used material for the synthesis of monolithic supports is poly(glycidyl methacrylate-co-ethylene dimethacrylate) [25]. Monoliths are chromatographic materials produced as a continuous phase, characterized by a unique structure made of a dense channel network [26,27]. They recently became particularly attractive for the isolation and purification of large biomolecules [27,28] and their use is possible even in the analytical field [26,27,29,30]. Usually, monoliths’ specific area is lower if compared to beads, but slightly higher than that of membranes.

An important advantage of both membranes and monoliths over chromatographic beads is represented by the fact that capacity and resolution for convective materials are independent of flow rate over a wide range of values [5,6,31,32,33,34,35,36,37,38,39] and this allows them to work at higher flow rates reducing process time. On the other hand, it is well-known that packed-bed column performance is strongly dependent on flow rate and that their binding capacity decreases increasing the flow rate [4,32,39,40].

Aside the type of matrix used, another important element that influences the performance of affinity chromatography is the affinity ligand, which is a molecule that has to be bound covalently to the surface of the base material and it has to be stable during the immobilization procedure as well as during the purification process. Among all types of ligands available for the purification of proteins, there are several dyes able to interact with these biomolecules: dyes are classified as affinity ligands, since they mimic the structure of coenzymes and enzyme cofactors, interacting with the active sites of proteins and enzymes [41,42,43,44]. Dye ligands, as triazine dyes, represent an important alternative to natural ligands [41,45,46,47]. Cibacron Blue F3GA (CB for simplicity) is a commonly used triazine dye affinity ligand for protein purification; in particular, it can specifically and reversibly interact with albumin [41,48,49,50,51,52,53]. CB was also successfully immobilized on the surface of membranes [54,55,56,57,58] and monoliths [59,60] for the recovery of other different biomolecules.

The present work aims at the development of a process for the selective recovery of bovine serum albumin (BSA), chosen as the model protein, through the characterization of CB-affinity materials. In particular, a commercially available CB resin (HiTrap^TM^ Blue HP column, GE Healthcare Life Sciences, Milan, Italy), regenerated cellulose membranes (Sartorius Stedim Biotech, Göettingen, Germany) and polymeric monoliths (Convective Interaction Media (CIM) discs, BIA Separations, Ljubljana, Slovenia) were employed as support materials. The surfaces of the membranes and monoliths were functionalized using CB-affinity ligand, developing and optimizing an appropriate experimental protocol. The performance of the three different chromatographic materials was evaluated in terms of BSA binding capacity at saturation, at 10% breakthrough and BSA productivity. The obtained results represent a relevant preliminary basis for the development and scale-up of the purification process that can be successfully based on the use of convective chromatographic materials.

## 2. Materials and Methods

### 2.1. Chemicals and Chromatographic Materials

BSA and all buffer chemicals were purchased from Sigma-Aldrich (Milan, Italy). Pure protein solutions were prepared daily by dissolving the appropriate amount of BSA in 0.1 M Tris-HCl pH 8.0 (for the resin), 0.05 M Tris-HCl containing 0.05 M NaCl pH 8.0 (for membranes) and 25 mM phosphate buffer containing 0.05 M NaCl pH 7.4 (for monoliths). These buffers were used as equilibration and washing buffers, while 0.05 M Tris-HCl containing 0.05 M NaCl and 0.5 M NaSCN pH 8.0 was used as elution buffer, for all supports. The producer of each material suggested the type of equilibration/washing buffers, as well as that of elution buffers to use during adsorption experiments.

Buffers and BSA solutions were filtered prior to use with 0.45 μm Sartorius filter made of cellulose nitrate.

Cibacron Blue F3GA (CB) is the ligand investigated in this study and it was purchased from Polysciences/Europe GmbH (Hirschberg an der Bergstrasse, Germany).

HiTrap^TM^ Blue HP columns, pre-packed with Blue Sepharose^TM^ High Performance, were purchased from GE Healthcare (Milan, Italy). Regenerated cellulose membranes were kindly provided by Sartorius Stedim Biotech GmbH (Göettingen, Germany) and have an average pore size of 0.45 μm. Convective Interaction Media (CIM) discs are the polymeric monolithic supports used in this work and were kindly provided by BIA Separations GesmbH (Ljubljana, Slovenia); monoliths are made of poly(glycidyl methacrylate-co-ethylene dimethacrylate) and were provided with epoxy functionality.

Geometric characteristics of all supports are summarized in Table 1: packed column and monolith dimensions were given by the respective producers, while membranes were cut into discs of fixed diameter and their thickness was measured using a micrometer (Absolute, Mitutoyo).

Pure protein concentration was measured by UV absorption at 280 nm using a UV-vis spectrophotometer (UV-1601 Shimadzu Italia, Milan, Italy).

### 2.2. Membrane Functionalization

**Figure 1:**
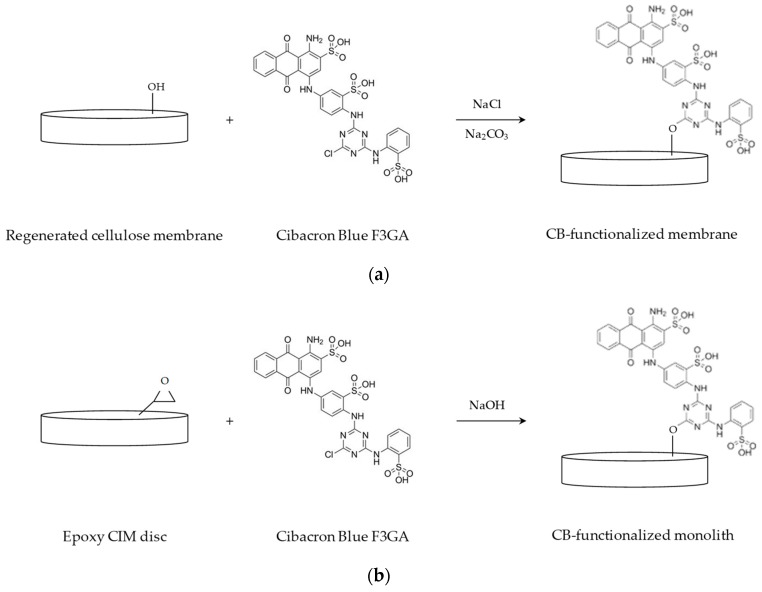
Cibacron Blue F3GA immobilization reaction on (**a**) regenerated cellulose membranes and (**b**) epoxy activated monoliths.

Affinity membranes were prepared by chemical immobilization of CB dye ligand on the surface of the convective supports, that were cut into discs of 2.6 cm diameter. Before modification, the membranes were equilibrated overnight in Phosphate Buffer Saline (PBS) at pH 7.0. A schematic representation of the CB immobilization reaction is shown in Figure 1a.

Ligand immobilization was performed incubating the membranes with a solution of 10 mg/mL of CB in water at 60 °C for 1 h, under stirring. This reaction was followed by the addition of 20% w/v NaCl aqueous solution; after 1 h, a solution of 25% w/v Na_2_CO_3_ was added to catalyze the reaction, carried out at 80 °C for 4 h [54,57,61].

The affinity membranes obtained were washed several times with hot water, 20% v/v methanol, 2 M NaCl aqueous solution, adsorption buffer, elution buffer, water, 20% v/v methanol and 2 M NaCl, in this order, until the unbound dye was completely removed. Membranes were stored at 4 °C in 0.05 M phosphate aqueous solution at pH 7.0 containing 0.02% sodium azide to prevent microbial growth [62].

CB-affinity ligand density was experimentally determined for a different number of modified membranes, following the procedure developed by Ruckenstein and Zeng [63]. CB membranes were hydrolyzed with 2 mL of 12 N hydrochloric acid for 30 min at 80 °C. The obtained solution was diluted to 6 N using distilled water and finally neutralized with 4 mL of 6 N NaOH aqueous solution [47]. Dye concentration in the final solution was determined by absorbance reading at 610 nm.

### 2.3. Monolith Functionalization

Before modification, monoliths were thoroughly washed with distilled water. The immobilization of the affinity ligand on the surface of the polymeric monoliths took place in recirculation mode, placing each monolith disc into a Plexiglas module, connected to a peristaltic pump (Miniplus 3, Gilson, Milan, Italy). A schematic representation of the CB immobilization reaction is shown in Figure 1b.

A total of 50 mL of 5 mg/mL CB solution containing 1 M NaOH was fed to the column at 80 °C for 3 h. After modification, the affinity monoliths were washed using distilled water and an aqueous solution of 20% methanol, to remove the unbound CB, and were stored at 4 °C in water containing 0.02% sodium azide to prevent microbial growth [62].

CB-ligand density on the surface of monoliths was not measured, since the technique adopted for membranes was disruptive. However, monoliths after modification were homogeneously colored in blue and the effectiveness of modification was proved by BSA adsorption tests.

### 2.4. Chromatographic Materials Characterization

Membranes, monoliths and the packed column were characterized in dynamic adsorption experiments, using pure BSA solutions. Dynamic binding capacity at saturation and at 10% breakthrough were calculated from the relevant breakthrough curves while productivity was derived from the mass of BSA eluted. A Fast Protein Liquid Chromatography (FPLC) and an AKTA Purifier 100 (GE Healthcare Life Sciences, Milan, Italy) were used to perform the dynamic characterization of all supports.

A summary of the dynamic experiments parameters is reported in Table 2.

Pure BSA solutions at different initial concentrations, from 0.25 to 1.4 mg/mL, were fed to the chromatographic materials that were then washed and eluted using the appropriate buffers (see Section 2.1). Membranes and monoliths were placed inside specific holders. In particular, a layered stack of 5 membrane discs of 2.6 cm diameter (due to the presence of o-rings inside the membrane holder, the effective diameter reduces to 2.2 cm) was placed into a stainless steel membrane holder, designed by our research group to ensure the correct flow distribution and the appropriate degree of membrane compression. For the case of monoliths, each CIM disc was placed inside a column holder, designed and provided by BIA Separations.

By combining different values of initial BSA concentration with different values of flow rate, a significant number of experiments were performed, using more than one set of modified membranes and monoliths. However, due to the limited amount of monoliths and packed columns available, the experiments were performed as single sets of measurements. For this reason a statistical analysis of the data obtained was not possible.

## 3. Results and Discussion

### 3.1. Membrane Ligand Density

The functionalization of regenerated cellulose membranes resulted in an average CB density of 120 mg/mL. This value is much higher than that characterizing the commercially available packed column used in this work, since the technical sheet of this stationary phase reports a ligand density of 4 mg of CB per mL of resin. However, the higher CB density on membranes does not reflect a higher capacity, probably because the ligands are not completely available. As far as the monolith is concerned, the ligand density was indirectly measured performing BSA binding experiments, to check the effectiveness of the modification procedure.

### 3.2. Dynamic Binging Capacity

A parameter expressing the performance of chromatographic materials is the dynamic binding capacity at saturation (*DBC*_100%_), defined by the following equation:

(1) $$DBC_{100\%}=\frac{m_{ads,100\%}}{V_{\mathrm{support}}}$$

where $m_{ads,100\%}$ is mass of product adsorbed performing the adsorption step until saturation and $V_{\mathrm{support}}$ is the volume of the chromatographic support.

However, due to the high product value and the high downstream processing costs, in the biopharmaceutical industry the adsorption step is usually stopped before column saturation, when the solute concentration in the stream exiting the chromatographic column reaches a particular value that usually corresponds to 10% breakthrough. In this case the parameter of interest to be calculated is the dynamic binding capacity at 10% breakthrough (*DBC*_10%_), that represents the amount of protein adsorbed per column volume, expressed by the following equation:

(2) $$DBC_{10\%}=\frac{m_{ads,10\%}}{V_{\mathrm{support}}}$$

where $m_{ads,10\%}$ is mass of product adsorbed performing the adsorption step until 10% breakthrough and $V_{\mathrm{support}}$ is the volume of the chromatographic media.

**Figure 2:**
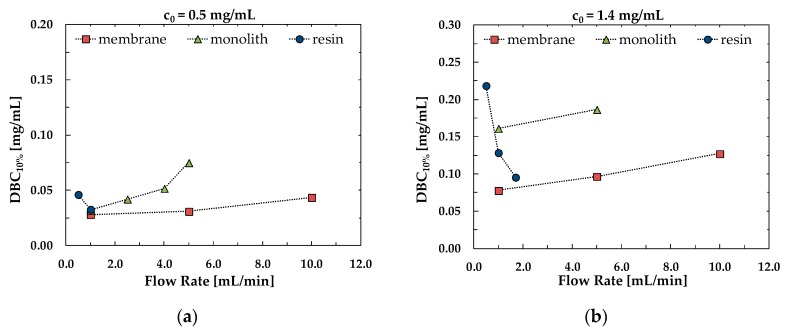
Comparison of dynamic binding capacity at 10% breakthrough (*DBC*_10%_) for the different media as a function of flow rate for initial BSA concentration of (**a**) 0.5 mg/mL and (**b**) 1.4 mg/mL.

The effect of flow rate on the values of dynamic binding capacity at 10% breakthrough is shown in Figure 2 for membranes and monoliths, as well as for packed columns, for two different values of BSA concentration in the feed. The packed column is the one whose performance is mostly and negatively influenced by the feed flow rate, since *DBC*_10%_ decreases as the flow rate increases, contrary to what happens for the convective materials. This means that in the case of a packed column, the capture process has to be performed at a low velocity, increasing process time but maintaining the capacity high.

**Figure 3:**
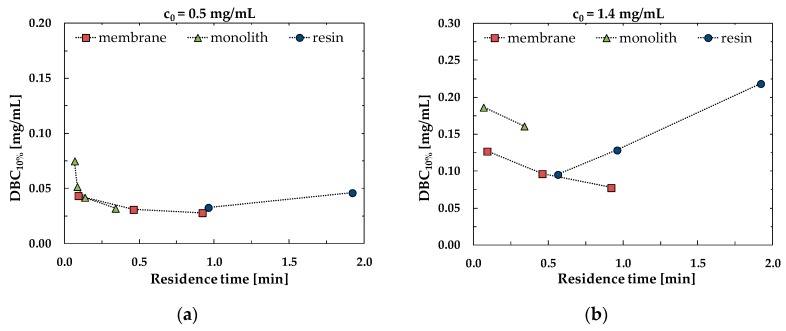
Comparison of *DBC*_10%_ for the different media as a function of residence time for initial bovine serum albumin (BSA) concentration of (**a**) 0.5 mg/mL and (**b**) 1.4 mg/mL.

The performance of the chromatographic materials studied can be also evaluated by plotting the dynamic binding capacity at 10% breakthrough against the residence time. From the plots shown in Figure 3 it can be concluded that membranes and monoliths give the same capacities as the resin but at lower residence time, demonstrating once again that the capture process is faster and more efficient using convective media.

**Figure 4:**
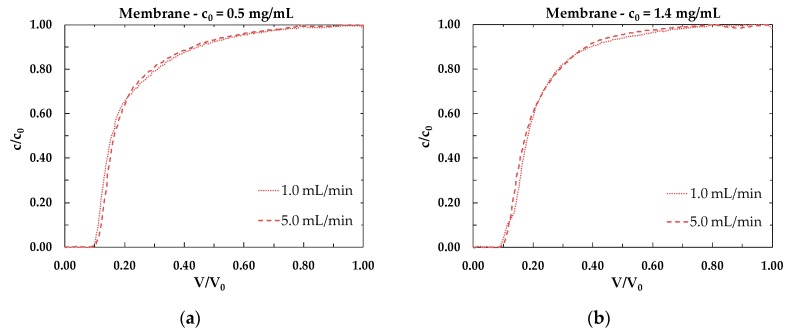

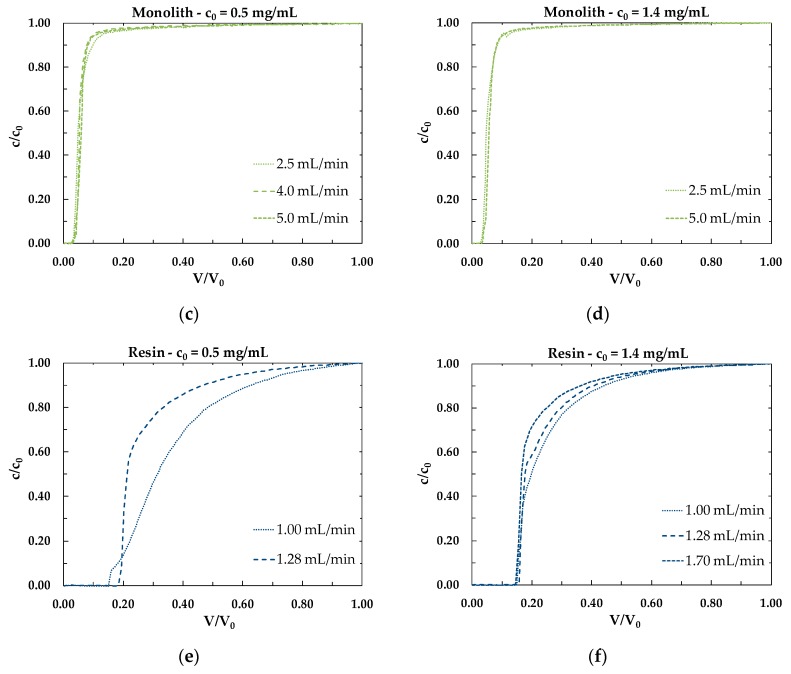
Experimental breakthrough curves as functions of flow rate for initial BSA concentration of 0.5 mg/mL and 1.4 mg/mL in the case of (**a**,**b**) membranes, (**c**,**d**) monoliths and (**e**,**f**) packed column.

The influence of flow rate on the shape of the breakthrough curves for all stationary phases studied can be observed in Figure 4. The most visible effect is noticeable for the packed column, Figure 4e,f: this result indicates that the resin is affected by non-negligible diffusional limitations, that influences the performance of the chromatographic material.

The data reported are the result of a single set of experiments. However, many chromatographic cycles were performed for each support, changing the initial BSA concentration and the flow rate; more data can be found in the Supplementary Material, Figures S1 and S2. The results reported here and in the Supplementary Material confirm the characteristic behavior of convective chromatographic materials, in that the performance of convective stationary phases is much less affected by flow rate than packed columns as indicated in the literature [5,6,31,32,33,34,35,36,37,38,39].

### 3.3. Productivity

Productivity was used as an alternative and more appropriate way to express the performance of a chromatographic support. In this paper, productivity, at 10% breakthrough, was calculated according to the following equation:

(3) $$P_{10\%}=\frac{m_{elu}}{V_{\mathrm{support}}\;t_{cycle}}$$

where $m_{elu}$ is the mass of product eluted from the support, $V_{\mathrm{support}}$ is the volume of the chromatographic media and $t_{cycle}$ is the duration of a complete chromatographic cycle.

**Figure 5:**
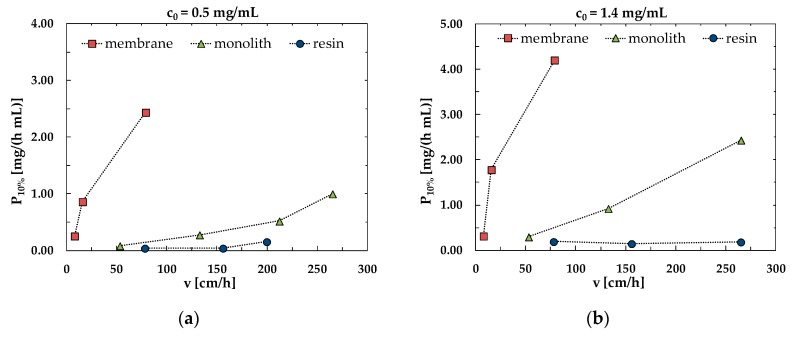
Comparison of the productivity at 10% breakthrough as a function of superficial velocity for an initial BSA concentration of (**a**) 0.5 mg/mL and (**b**) 1.4 mg/mL.

For each material studied, productivity was plotted against the superficial velocity, at fixed concentration. The results are reported in Figure 5.

The plots in Figure 5a,b clearly show that a higher productivity can be obtained with membranes and monoliths with respect to the packed column, when working at the same superficial velocity. Remarkably, the increase of productivity with increasing velocity is very limited for the resin if compared to membranes and monoliths.

## 4. Conclusions

An experimental study regarding BSA capture was performed through affinity chromatography using different chromatographic media functionalized with Cibacron Blue F3GA ligand. Resins, membranes and monoliths were characterized and their performance compared in terms of binding capacity and productivity. The preliminary results obtained demonstrate that the dynamic binding capacity at 10% breakthrough is independent on flow rate in the case of membranes and monoliths. Thus, convective stationary phases were not affected by kinetic limitations in the range of superficial velocities investigated. Additionally, a strong effect of flow rate was observed for the packed bed column. According to this, the shape of the breakthrough curve was greatly affected by the increase of flow rate in the case of the packed column. At the industrial level, the capture step is usually performed until the concentration of the protein to recover reaches 10% breakthrough in the stream exiting the column. Therefore, according to the preliminary set of experiments performed, membranes and monoliths perform better than the packed column, since they can achieve higher capacities at lower residence times, speeding up the purification process. Similar conclusions are drawn from the results obtained in terms of productivity: at fixed initial BSA concentration and at fixed superficial velocity a productivity from 10 to 20 times higher than the packed column can be achieved using membranes.

The results shown demonstrate that the use of convective stationary phases can reduce the purification process time significantly, thus reducing buffer consumption and avoiding protein degradation. Moreover, the use of convective stationary phases can overcome packing requirements, as well as Clean In Place (CIP) and re-validation procedures.

The interesting flow behavior of convective materials is confirmed by the experimental investigation carried out and the obtained results can be used as a solid base for process scale-up and development. Indeed, these properties explain the enormous interest that the bioprocess industry has for membrane chromatography which enables a fast and efficient purification of large biomolecules such as viral vectors used in gene and cell therapies.

## Figures and Tables

**Table 1:** Diameter and height of the chromatographic supports.

Packed Column		Membrane		Monolith	
*d* (cm)	*h* (cm)	*d* (cm)	*h* (cm)	*d* (cm)	*h* (cm)
0.7	2.5	2.6	0.0240	1.2	0.3

**Table 2:** Flow rate (*F*) and corresponding superficial velocity (*v*) and residence time (*τ*) for each chromatographic support.

Packed Column			Membrane			Monolith		
*F* (mL/min)	*v* (cm/h)	*τ* (min)	*F* (mL/min)	*v* (cm/h)	*τ* (min)	*F* (mL/min)	*v* (cm/h)	*τ* (min)
0.5	78.0	1.92	0.5	7.9	0.92	1.0	53.1	0.34
1.0	155.9	0.96	1.0	15.8	0.46	2.5	132.6	0.14
1.2	199.6	0.75	5.0	78.9	0.09	4.0	212.2	0.09
1.7	265.0	0.56	10.0	157.8	0.05	5.0	265.3	0.07

## Supplementary Materials

The following are available online at https://www.mdpi.com/2077-0375/10/1/1/s1, Figure S1: *DBC*_10%_ as a function of flow rate at fixed initial BSA concentration for (**a**) resin, (**b**) membrane and (**c**) monolith. Each point in the plots represent a chromatographic experiment. All the data presented were obtained without considering the dispersion contributions, that take into account for the system dead volume; for this reason, the values of *DBC*_10%_ are higher than those reported in the paper (please, refer to Figure 2 of the main manuscript), Figure S2: Breakthrough curves at fixed flow rate, as a function of initial BSA concentration for (**a**) resin, (**b**) membrane and (**c**) monolith.
