# Supplementary material for: Affinity Membranes and Monoliths for Protein Purification
Source: Membranes (Basel). 2019 Dec 24;10(1):1. doi: 10.3390/membranes10010001 (PMC7022333; doi:10.3390/membranes10010001)
Supplement: Supplementary file 1 [file membranes-10-00001-s001.pdf]

membranes

Supporting Information

Affinity Membranes and Monoliths for Protein Purification

Eleonora Lalli, Jouciane S. Silva, Cristiana Boi *and Giulio C. Sarti

Dipartimento di Ingegneria Civile, Chimica, Ambientale e dei Materiali, DICAM, Alma Mater Studiorum Università di Bologna, via Terracini 28, 40131, Bologna, Italy; eleonora.lalli2@unibo.it (E.L.); jouciane@gmail.com (J.S.S.); giulio.sarti@unibo.it (G.C.S.)

* Correspondence: cristiana.boi@unibo.it; Tel.: +39 051 20 90 432

Data related to dynamic binding capacity at 10% breakthrough ($DBC_{10\%}$) are shown in Figure S1. The three plots, associated to the different chromatographic supports used, represent the trend of $DBC_{10\%}$ as a function of flow rate, at different values of initial BSA concentration.

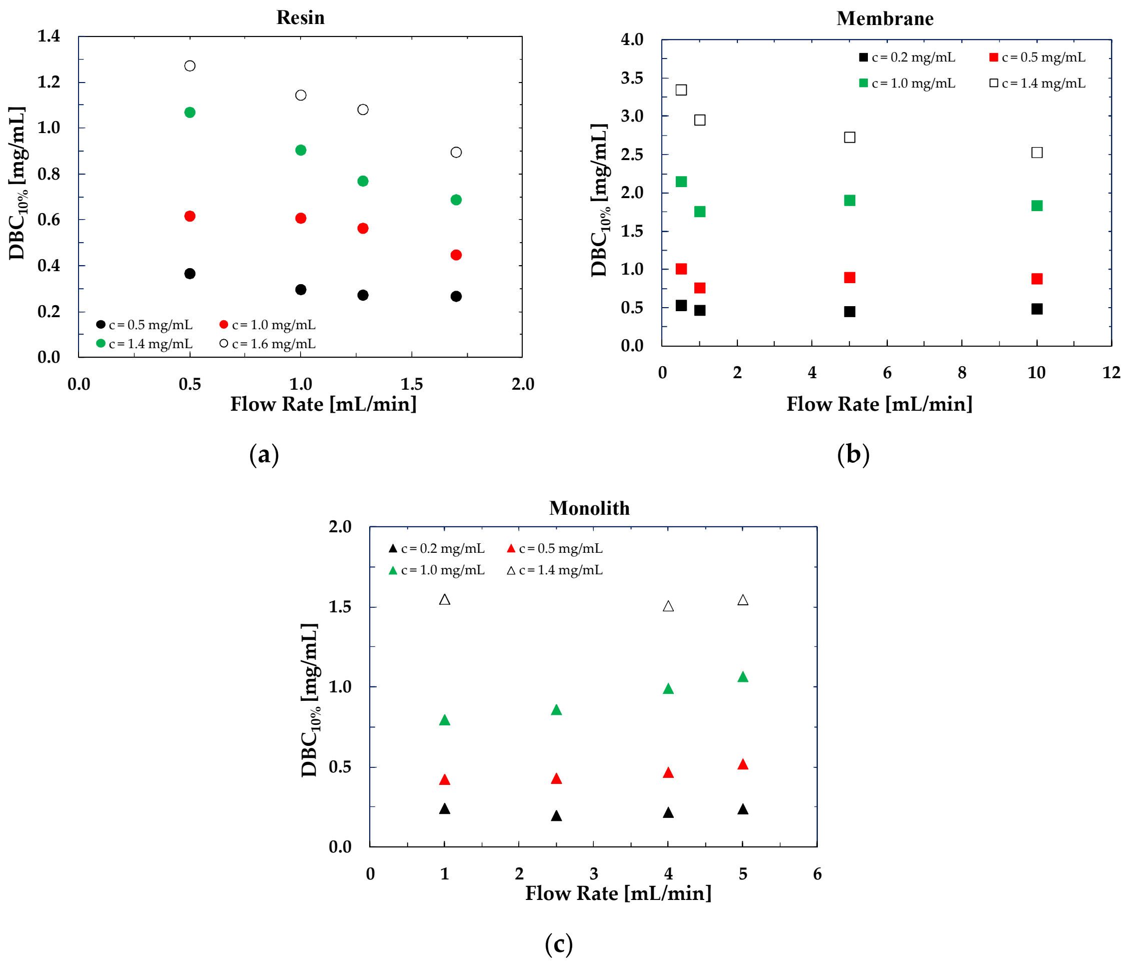

Figure S1. $DBC_{10\%}$ as a function of flow rate at fixed initial BSA concentration for (**a**) resin, (**b**) membrane and (**c**) monolith. Each point in the plots represent a chromatographic experiment. All the data presented were obtained without considering the dispersion contributions, that take into account for the system dead volume; for this reason, the values of $DBC_{10\%}$ are higher than those reported in the paper (please, refer to Figure 2 of the main manuscript).

Examples of breakthrough curves as a function of initial BSA concentration are presented in Figure S2, for the three chromatographic supports characterized at a fixed flow rate value.

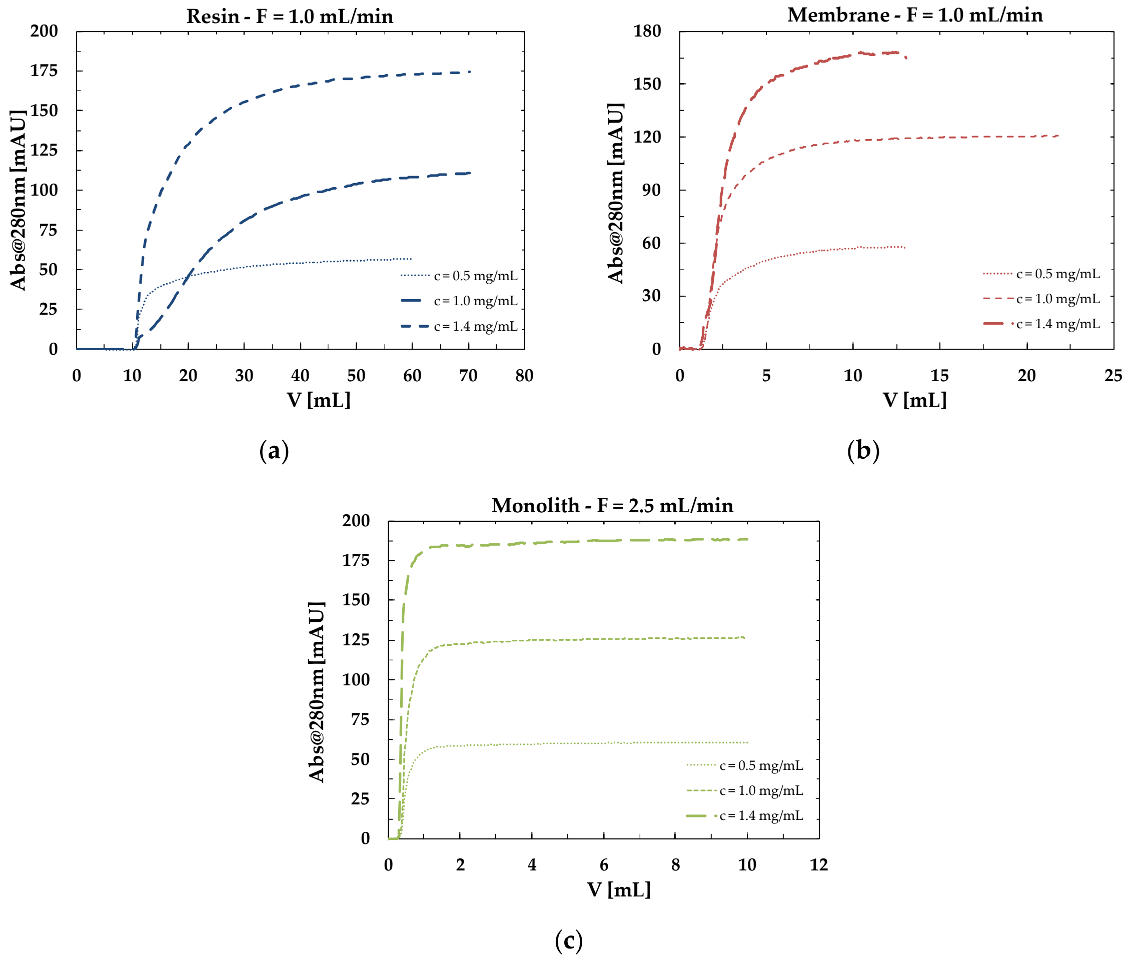

Figure S2. Breakthrough curves at fixed flow rate, as a function of initial BSA concentration for (**a**) resin, (**b**) membrane and (**c**) monolith.

© 2019 by the authors. Submitted for possible open access publication under the terms and conditions of the Creative Commons Attribution (CC BY) license (http://creativecommons.org/licenses/by/4.0/).
